# Acute and chronic neuropathic pain profiles after video-assisted thoracic surgery: A prospective study

**DOI:** 10.1097/MD.0000000000019629

**Published:** 2020-03-27

**Authors:** Shiho Takenaka, Ayano Saeki, Norihiko Sukenaga, Ryusuke Ueki, Nobutaka Kariya, Tsuneo Tatara, Munetaka Hirose

**Affiliations:** a Department of Anesthesiology and Pain Medicine, Hyogo College of Medicine, Hyogo; b Anesthesia Department, Cancer Pain Clinic, Fukuyama City Hospital, Fukuyama, Japan.

**Keywords:** DN4, neuropathic pain, postoperative pain, thoracic surgery

## Abstract

Acute postsurgical pain, probably including acute neuropathic pain (ANeP), starts at the early postoperative period, and chronic postsurgical pain including chronic neuropathic pain (CNeP) persists at least 3 months after surgery. Although it must be important for prevention and treatment of acute and chronic postoperative pain to reveal the time course of postoperative neuropathic characteristics, a neuropathic pain profile after surgery has not been evaluated.

Pain status at the surgical site in adult patients who underwent video-assisted thoracic surgery (VATS) for lung cancer was prospectively assessed until 12 months after surgery. Neuropathic characteristics were assessed using the Douleur Neuropathique 4 (DN4) questionnaire until 6 days after surgery and the DN2 questionnaire throughout the study.

Twenty-seven patients were enrolled in this study. Pain intensity at surgical sites were significantly higher at 1 and 6 days after surgery during resting state, and were also significantly higher at 3, 6, and 12 months after surgery during movement than those before surgery. The incidence of ANeP was 33.3% at 1 day, and 18.5% at 6 days after surgery. The incidence of CNeP decreased to 12.5% at 3 months, 5.0% at 6 months, and 0.0% at 12 months after surgery. The number of neuropathic characteristics, assessed by DN2 scores, significantly increased at 1 and 6 days after surgery, compared to those before surgery. DN2 scores at 3, 6, and 12 months after surgery, however, showed no significant differences compared to those before surgery.

In patients with acute postsurgical pain, 20% to 30% of patients show ANeP characteristics, and the incidence of CNeP gradually decreases after VATS in patients with chronic postsurgical pain.

## 1 Introduction

Acute postsurgical pain increases postoperative complications in the early postoperative period, and chronic postsurgical pain, which includes chronic neuropathic pain (CNeP),^[1,2]^ persists at least 3 months after surgery, deteriorating the quality of life in patients after surgery. Recently, acute neuropathic pain (ANeP), which may start after trauma at the somatosensory nerve system, likely emerges in the early postoperative period.^[3–5]^ Since mechanisms of transition from acute to chronic postsurgical pain is thought to include the development of ANeP and CNeP,^[2,4,5]^ it must be important for prevention and treatment of acute and chronic postsurgical pain to understand the time course of neuropathic characteristics after surgery. A neuropathic pain profile after surgery, however, has not been evaluated. In the present study, to reveal the neuropathic pain profiles after surgery, we prospectively examined the time course of the Douleur Neuropathique 4 (DN4) and DN2 questionnaire scores, which were developed to screen for neuropathic pain,^[3,6]^ in patients undergoing video-assisted thoracic surgery (VATS).

## 2 Methods

This study was approved by the Ethics Committee of the Hyogo College of Medicine (#0239) and was registered in the UMIN Clinical Trials Registry (UMIN000014908).

### 2.1 Population

A total of 27 patients with lung cancer who were scheduled for VATS were enrolled in this prospective observational study. Written informed consent was obtained from all participants who met the following criteria: age over 20 years, American Society of Anesthesiologists (ASA) physical status I to III, scheduled to undergo elective VATS. Exclusion criteria included previous thoracic surgery, presence of a psychiatric or neurologic disorder, chronic pain at the other sites of the incision or related areas of the surgery, and liver or renal dysfunction.

### 2.2 Perioperative management

The patients did not receive premedication. Dexamethasone 3.3 mg was intravenously injected for prevention of postoperative nausea and vomiting before induction of anesthesia. General anesthesia was induced with propofol, fentanyl, and rocuronium, with a continuous infusion of remifentanil, followed by insertion of a double-lumen endotracheal tube, and was maintained with a continuous infusion of remifentanil and an intermittent injection of fentanyl and rocuronium, with a target-controlled infusion of propofol. The dose of remifentanil was adjusted to maintain mean blood pressure within the range of ±20% of the pre-anesthesia level. Bispectral index was maintained between 40 and 60 by adjusting the target concentration of propofol administered during surgery. Intravenous fentanyl was administered up to a total dose of 4 to 5 μg·kg^−1^during general anesthesia. Rocuronium bromide was used as needed for muscle relaxation during surgery. The only regional anesthesia method used was intercostal nerve block, obtained by injection of 20 mL of 0.25% levobupivacaine administered by the surgeon at the incision site just before the surgical wound was closed with sutures. After surgery, a continuous intravenous infusion of 25 to 50 μg·h^−1^ of fentanyl was routinely administered until postoperative day 1. Intravenous flurbiprofen or acetaminophen was used for rescue analgesia.

### 2.3 Perioperative pain assessments

Pain intensity was evaluated using a Numerical Rating Scale (NRS). NRS, which comprises assessment using a 0 to 10 points scale, was used to assess pain intensity both at rest and during movement at surgical sites. The lowest value (0) was labeled ‘no pain’ and the highest value (10) was labeled ‘worst imaginable pain’. We used the DN4 and DN2 questionnaires to discriminate neuropathic pain from other pain states at surgical sites.^[6,7]^ The DN4 questionnaire evaluates 10 items: characteristics of pain (burning (1), painful cold (2), electric shocks (3)), symptoms in the region of pain (tingling (4), pins and needles (5), numbness (6), itching (7)), localized pain (hypoesthesia to touch (8), hypoesthesia to pricking (9)) and pain caused or increased by brushing in the painful area (10). Items from #1 to #7 of the DN4 questionnaire are answered by interviewing patients, and items from #8 to #10 require examination of patients. DN4 score is a total count of these 10 items existing in each patient, and the cut-off value for the diagnosis of neuropathic pain is a score of 4/10.^[6,7]^ Additionally, DN2 score is a total count of 7 items including 3 pain characteristics and 4 pain symptoms, and the cut-off value for the diagnosis of neuropathic pain is a score of 3/7.^[6]^ DN2 questionnaires can also be applied to screen neuropathic pain only by interviewing, instead of DN4 questionnaires.^[3,6]^ The incidences of ANeP and CNeP were calculated by the ratio between the number of patients whose DN4/DN2 scores were 3/4 or more and the total number of patients having with acute or chronic postsurgical pain. The same investigator interviewed all patients for evaluation of NRS, DN4, and DN2 before surgery (T0), and at 1 (T1) and 6 days (T2) after surgery, sending NRS and DN2 questionnaires and collecting the responses by mail at 3 (T3), 6 (T4), and 12 months (T5) after surgery.

### 2.4 Sample size calculation

The sample size was calculated using software (PS Power and Sample Size Calculations, version 3.0, Dupont WD and Plummer WD). The calculation was done based on the assumption that a type I error probability of 0.01 (0.05/5 = 0.01) as 5 parameters at T1, T2, T3, T4, and T5 were tested, and power of 0.8. From a previous study,^[8]^ a standard deviation of DN4 values was 1.6, and a difference in DN4 values before and after surgery was 2. Then, the estimated sample size was 11.

### 2.5 Statistics

All statistical testing was performed using JMS Pro version 13.1.0 (SAS Institute Inc, Cary, NC). The Kruskal–Wallis test, followed by the Wilcoxon test with Bonferroni correction, was used to compare the time course of changes in pain status. A statistically significant level after a Bonferroni adjustment was considered as ^∗^*P* < .0125 and ^∗∗^*P* < .0025 vs T0 when 5 parameters at T1, T2, T3, T4, and T5 were tested (0.05/5 = 0.01, 0.01/5 = 0.002).

## 3 Results

### 3.1 Perioperative changes in pain intensity

**Figure 1:**
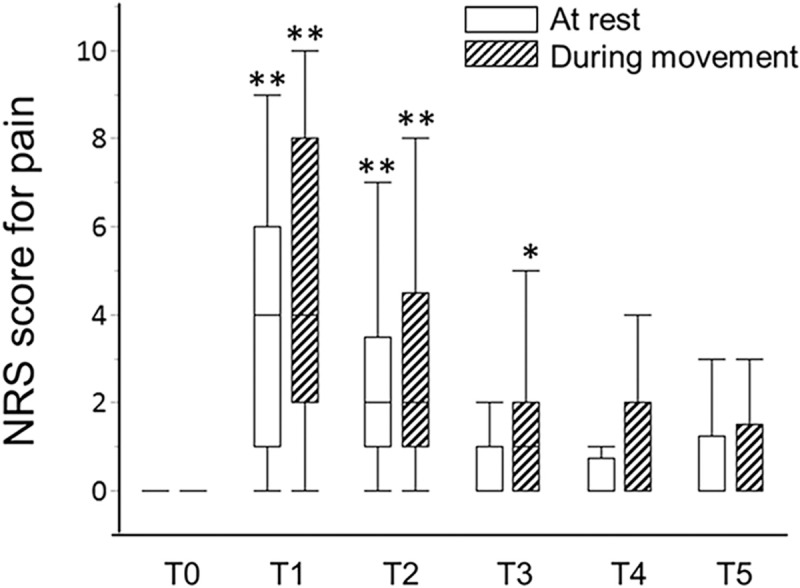
Box- and whisker dot plots showing perioperative changes in pain intensity at the surgical site. NRS = numerical rating scale, T0 = 1 day before surgery, T1 = 1 day after surgery, T2 = 6 days after surgery, T3 = 3 months after surgery, T4 = 6 months after surgery, T5 = 12 months after surgery. Statistically significant levels after a Bonferroni adjustment were considered as ^∗^*P* < .01 and ^∗∗^*P* < .002 vs T0.

Table 1 shows the patients’ demographics. No patients received an intraoperative conversion from VATS to open thoracic surgery. The duration of surgery was 138.3 ± 48.4 min. Five of 27 patients (18.5%) had preoperative pain at surgical sites. At T1 and T2, 25 of 27 (92.6%) and 23 of 27 (85.2%) patients had acute postsurgical pain. Chronic postsurgical pain was observed in 15 of 24 (62.5%) at T3, 11 of 20 (55.0%) at T4, and 5 of 14 (35.7%) at T5 (Table 2). Pain intensity at surgical sites, evaluated using NRS, were significantly higher at T1 and T2 during resting state, and were significantly higher at T1, T2, and T3 during movement than those at T0 (Fig. 1).

**Table 1:**
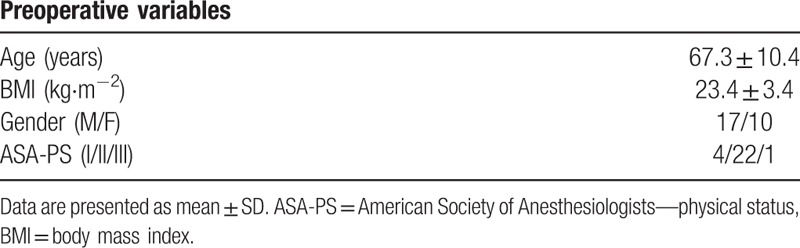
Patient demographics.

**Table 2:**
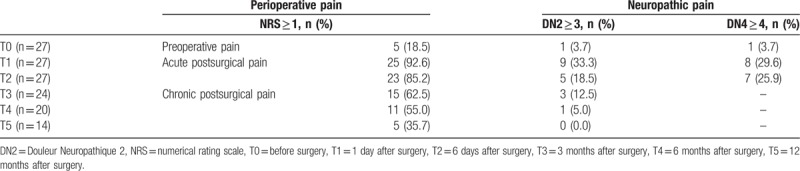
The incidence of perioperative pain and the ratio of neuropathic pain.

### 3.2 Perioperative changes in neuropathic pain characteristics

**Figure 2:**
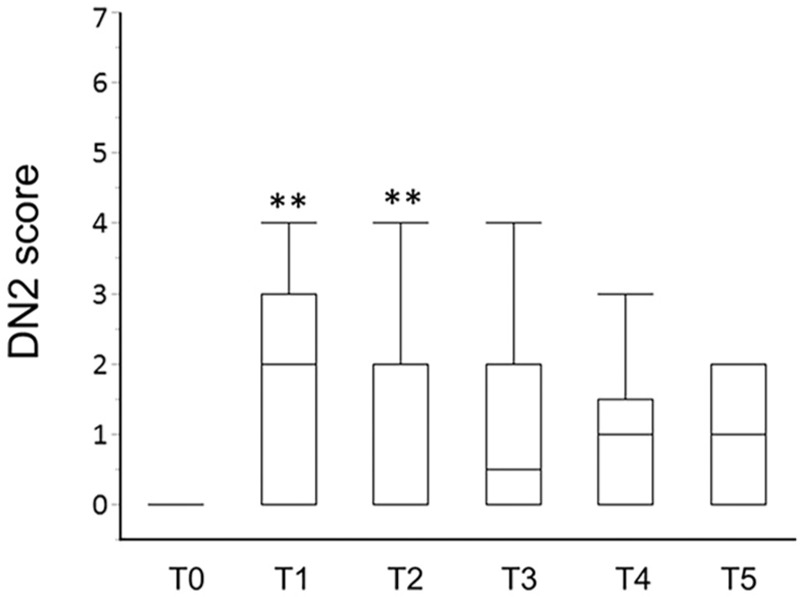
Box- and whisker dot plots showing perioperative changes in DN2 score. DN2 = Douleur Neuropathique 2. T0 = 1 day before surgery, T1 = 1 day after surgery, T2 = 6 days after surgery, T3 = 3 months after surgery, T4 = 6 months after surgery, T5 = 12 months after surgery. Statistically significant levels after a Bonferroni adjustment were considered as ^∗^*P* < .01 and ^∗∗^*P* < .002 vs T0.

Of the 27 patients, one patient (3.7%) showed neuropathic characteristics at T0, and 33.3% and 29.6% patients at T1 and T2 showed ANeP characteristics, evaluated by the DN2. The incidence of ANeP was 29.6% and 25.9% at T1 and T2, when the DN4 was applied to evaluate neuropathic profile. At T3, T4, and T5, 12.5%, 5.0%, and 0.0% patients showed CNeP characteristics, evaluated by the DN2 (Table 2). All 3 patients, who showed CNeP at T3, already had neuropathic characteristics of ANeP at T1. In Figure 2, DN2 scores at T1 and T2 were significantly higher than those at T0. DN2 scores at T3, T4, and T5, however, showed no significant differences compared to those at T0 (Fig. 2).

## 4 Discussion

This is the first study revealing the time course change of neuropathic pain characteristics, including both ANeP and CNeP, assessed by the DN4 or DN2 questionnaires before and after thoracic surgery for 1 year.^[5,8,9]^ The incidence of acute postsurgical pain was around 90%, and that of chronic postsurgical pain was 40% to 60% in patients undergoing VATS in the present study. The incidence and severity of acute and chronic postsurgical pain in patients undergoing thoracic surgery is reportedly relatively high compared to other surgeries.^[10,11]^ In contrast, the ratio of ANeP in patients with acute postsurgical pain was 20% to 30% at the early postoperative period, and that of CNeP in patients with chronic postsurgical pain was 12.5% at 3 months after surgery, which decreased to 0.0% at 12 months after VATS in the present study. ANeP characteristics in the DN4/DN2 questionnaire showed significant increases at 1 and 6 days after surgery, concurrent with the occurrence of acute postsurgical pain. Boloeil et al investigated patients undergoing several types of surgery using the DN4 questionnaire, documenting that the incidence of ANeP was 5.6% of patients on the day of surgery, and 12.9% of patients at 2 days after surgery.^[3]^ As this previous study included a wide range of surgical procedures (e.g., orthopedic, abdominal, ear nose throat, urology, or neurosurgery), some of which may not cause trauma at the somatosensory nerve system. It is plausible that the incidence of ANeP after VATS in the present study was higher than that in the previous study.

There were some differences in the incidences of ANeP, which were 29.6% and 33.3% at T1, and 25.9% and 18.5% at T2 assessed by the DN2 and DN4 questionnaires, respectively in the present study. The DN2 questionnaires do not include three somatosensory examinations for hypoesthesia to touch, hypoesthesia to pricking, and pain caused or increased by brushing, which are the items #8 to #10 in the DN4 questionnaires. Since the degree of somatosensory disturbances caused by surgery changes daily from 1 day to 6 days after surgery, this assessment variance between DN4 and DN2 might affect the results of incidence of ANeP.

Intercostal nerve injury, which is believed to be one of the pathogenic factors of neuropathic pain after thoracic surgery, causes CNeP, of which incidence was reportedly between 23% and 66%.^[12–14]^ Although Boloeil et al reported that ANeP increases the risk of CNeP,^[3]^ the incidence of CNeP in the present study was relatively lower than that in the previous reports. Preoperative risk factors for chronic postsurgical pain reportedly include preoperative pain and a history of psychological disorders.^[2]^ Since our exclusion criteria included preoperative chronic pain and presence of a psychiatric disorder, the decreased incidence of CNeP might be explained by preoperative factors rather than ANeP in the present study.

A limitation of this study is the small number of patients enrolled. Further study is needed to evaluate preoperative risk factors for the development of ANeP and CNeP after surgery in a larger number of participants having with a psychiatric or neurologic disorder and preoperative chronic pain in a multi-institutional study.

## 5 Conclusions

The incidence of ANeP was 20% to 30% at the early postoperative period, and that of CNeP gradually decreased after VATS.

## Author contributions

**Conceptualization:** Munetaka Hirose.

**Data curation:** Shiho Takenaka, Ayano Saeki

**Investigation:** Shiho Takenaka, Ayano Saeki

**Methodology:** Shiho Takenaka, Ayano Saeki.

**Project administration:** Munetaka Hirose, Shiho Takenaka, Ayano Saeki.

**Supervision:** Norihiko Sukenaga, Ryusuke Ueki, Nobutaka Kariya

**Validation:** Ryusuke Ueki, Nobutaka Kariya, Tsuneo Tatara

**Writing – original draft:** Munetaka Hirose, Shiho Takenaka, Ayano Saeki.

**Writing – review & editing:** Munetaka Hirose.

Munetaka Hirose: 0000-0003-1291-2827.

Munetaka Hirose orcid: 0000-0003-1291-2827.
